# Supplementary material for: Estimating the global cost of vision impairment and its major causes: protocol for a systematic review
Source: BMJ Open. 2020 Sep 6;10(9):e036689. doi: 10.1136/bmjopen-2019-036689 (PMC7476478; doi:10.1136/bmjopen-2019-036689)
Supplement: Supplementary data [file bmjopen-2019-036689supp002.pdf]

ANNEX 2 - SEARCH STRATEGIES

MEDLINE (Ovid)

1. "Global Burden of Disease"/
2. "costs and cost analysis"/
3. cost-benefit analysis/
4. "cost of illness"/
5. health care costs/
6. "Health Services Needs and Demand"/ec, sn [Economics, Statistics & Numerical Data]
7. Health Care Surveys/ec, sn [Economics, Statistics & Numerical Data]
8. Health Expenditures/ec, sn [Economics, Statistics & Numerical Data]
9. Health Resources/ec, sn [Economics, Statistics & Numerical Data]
10. Global Health/ec, sn [Economics, Statistics & Numerical Data]
11. ((global or economic) adj2 burden).tw.
12. ((cost or costs) adj2 (benefit or analysis or illness or direct or indirect or Intangible)).tw.
13. Efficiency/
14. Absenteeism/
15. Presenteeism/
16. productivity.tw.
17. "Severity of Illness Index"/ec [Economics]
18. Employment/ec [Economics]
19. Sick Leave/ec, sn [Economics, Statistics & Numerical Data]
20. (absenteeism or presenteeism or productivity).tw.
21. Caregivers/ec, sn [Economics, Statistics & Numerical Data]
22. or/1-21
23. exp eye diseases/
24. exp vision disorders/
25. ((vision or visual$) adj2 (impair$ or loss or disorder)).tw.
26. (cataract$ or glaucoma or macula$ degeneration).tw.
27. (diabetic retinopathy or refractive error$ or trachoma or corneal opacity).tw.
28. or/23-27
29. 22 and 28
30. limit 29 to yr="2000 -Current"

CRD database

The CRD database will be searched using the following MeSH terms:

MeSH DESCRIPTOR Eye Diseases EXPLODE ALL TREES

MeSH DESCRIPTOR Vision Disorders EXPLODE ALL TREES
