# Supplementary material for: Estimating the global cost of vision impairment and its major causes: protocol for a systematic review
Source: BMJ Open. 2020 Sep 6;10(9):e036689. doi: 10.1136/bmjopen-2019-036689 (PMC7476478; doi:10.1136/bmjopen-2019-036689)
Supplement: Author's manuscript [file bmjopen-2019-036689.draft_revisions.pdf]

BMJ Open is committed to open peer review. As part of this commitment we make the peer review history of every article we publish publicly available.

When an article is published we post the peer reviewers' comments and the authors' responses online. We also post the versions of the paper that were used during peer review. These are the versions that the peer review comments apply to.

The versions of the paper that follow are the versions that were submitted during the peer review process. They are not the versions of record or the final published versions. They should not be cited or distributed as the published version of this manuscript.

BMJ Open is an open access journal and the full, final, typeset and author-corrected version of record of the manuscript is available on our site with no access controls, subscription charges or pay-per-view fees (http://bmjopen.bmj.com).

If you have any questions on BMJ Open's open peer review process please email info.bmjopen@bmj.com

BMJ Open

Estimating the global cost of Vision Impairment and its major causes: protocol for a systematic review

Journal:	*BMJ Open*
Manuscript ID	bmjopen-2019-036689
Article Type:	Protocol
Date Submitted by the Author:	27-Dec-2019
Complete List of Authors:	Marques, Ana Patricia; London School of Hygiene and Tropical Medicine International Centre for Eye Health,<br>Ramke, Jacqueline; London School of Hygiene and Tropical Medicine, International Centre for Eye Health; University of Auckland, School of Optometry and Vision Science<br>Cairns, John; London School of Hygiene and Tropical Medicine, Department of Health Services Research and Policy<br>Butt, Thomas; University College London, UCL Institute of Ophthalmology<br>Zhang, Justine; London School of Hygiene and Tropical Medicine Faculty of Infectious and Tropical Diseases, International Centre for Eye Health, Clinical Research Department<br>Faal, Hannah; University of Calabar, Department of Ophthalmology<br>Taylor, Hugh; University of Melbourne School of Population and Global Health,<br>Jones, Iain; Sightsavers<br>Congdon, Nathan; Queen's University, Centre for Public Health; Sun Yat-Sen University Zhongshan Ophthalmic Center<br>Bastawrous, Andrew; London School of Hygiene and Tropical Medicine, International Centre for Eye Health<br>Braithwaite, Tasanee; London School of Hygiene and Tropical Medicine, International Centre for Eye Health; Moorfields Eye Hospital<br>Jovic, Marty; PricewaterhouseCoopers<br>Resnikoff, Serge; University of New South Wales, Brien Holden Vision Institute<br>Nandakumar, Allayala; Brandeis University Heller School for Social Policy and Management<br>Khaw, Peng; NIHR Biomedical Research Centre at Moorfields Eye Hospital and UCL Institute of Ophthalmology<br>Bourne, Rupert; Anglia Ruskin University, Vision and Eye Research Unit<br>Gordon, Iris; London School of Hygiene and Tropical Medicine, International Centre for Eye Health<br>Frick, Kevin; Johns Hopkins University Carey Business School - Baltimore Campus<br>Burton, Matthew J; London School of Hygiene and Tropical Medicine, International Centre for Eye Health; Moorfields Eye Hospital
Keywords:	OPHTHALMOLOGY, PUBLIC HEALTH, HEALTH ECONOMICS

I, the Submitting Author has the right to grant and does grant on behalf of all authors of the Work (as defined in the below author licence), an exclusive licence and/or a non-exclusive licence for contributions from authors who are: i) UK Crown employees; ii) where BMJ has agreed a CC-BY licence shall apply, and/or iii) in accordance with the terms applicable for US Federal Government officers or employees acting as part of their official duties; on a worldwide, perpetual, irrevocable, royalty-free basis to BMJ Publishing Group Ltd ("BMJ") its licensees and where the relevant Journal is co-owned by BMJ to the co-owners of the Journal, to publish the Work in this journal and any other BMJ products and to exploit all rights, as set out in our licence.

The Submitting Author accepts and understands that any supply made under these terms is made by BMJ to the Submitting Author unless you are acting as an employee on behalf of your employer or a postgraduate student of an affiliated institution which is paying any applicable article publishing charge ("APC") for Open Access articles. Where the Submitting Author wishes to make the Work available on an Open Access basis (and intends to pay the relevant APC), the terms of reuse of such Open Access shall be governed by a Creative Commons licence – details of these licences and which Creative Commons licence will apply to this Work are set out in our licence referred to above.

Other than as permitted in any relevant BMJ Author's Self Archiving Policies, I confirm this Work has not been accepted for publication elsewhere, is not being considered for publication elsewhere and does not duplicate material already published. I confirm all authors consent to publication of this Work and authorise the granting of this licence.

For peer review only

ESTIMATING THE GLOBAL COST OF VISION IMPAIRMENT AND ITS MAJOR CAUSES: PROTOCOL FOR A SYSTEMATIC REVIEW

Authors:

Ana Patricia Marques[1], Jacqueline Ramke[1,2], John Cairns[3], Thomas Butt[4] , Justine H. Zhang[1,5], Hannah Faal[6], Hugh Taylor[7], Iain Jones[8], Nathan Congdon[9,10], Andrew Bastawrous[1], Tasanee Braithwaite[1,11], Marty Jovic[12], Serge Resnikoff[13], Allyala Nandakumar[14], Peng T. Khaw[15], Rupert Bourne[16], Iris Gordon[1], Kevin Frick[17], Matthew J. Burton[1,11]

Affiliations:

1. International Centre for Eye Health, London School of Hygiene and Tropical Medicine, London, United Kingdom
2. School of Optometry and Vision Science, University of Auckland, Auckland, New Zealand
3. Health Services Research and Policy, London School of Hygiene and Tropical Medicine, London, United Kingdom
4. UCL Institute of Ophthalmology, University College London, London, United Kingdom
5. Manchester Royal Eye Hospital, Manchester, United Kingdom
6. Department of Ophthalmology, University of Calabar, Calabar, Nigeria
7. Indigenous Eye Health Unit, Melbourne School of Population and Global Health, The University of Melbourne, Melbourne, Australia
8. Sightsavers, Haywards Heath, United Kingdom
9. Centre for Public Health, Queen's University, Belfast, United Kingdom
10. Zhongshan Ophthalmic Center, Sun Yat-sen University, Guangzhou, China
11. Moorfields Eye Hospital, London, United Kingdom
12. PricewaterhouseCoopers, Sydney, Australia
13. Brien Holden Vision Institute and SOVS, University of New South Wales, Sydney, NSW, Australia
14. Heller School for Social Policy and Management, Brandeis University, Waltham, Massachusetts, United States of America
15. National Institute for Health Research Biomedical Research Centre, Moorfields Eye Hospital NHS Foundation Trust and UCL Institute of Ophthalmology, London, United Kingdom
16. Vision and Eye Research Institute, School of Medicine, Anglia Ruskin University, Cambridge, United Kingdom
17. Johns Hopkins Carey Business School, Baltimore, Maryland, United States of America

For peer review only - http://bmjopen.bmj.com/site/about/guidelines.xhtml

Corresponding author: Ana Patricia Marques

Address: International Centre for Eye Health, London School of Hygiene and Tropical Medicine, Keppel Street, London, WC1E 7HT

E-mail: Patricia.Marques@lshtm.ac.uk

E-mail addresses of all co-authors are available from the corresponding author upon reasonable request.

Word Count: ~2100

ABSTRACT

Introduction

Vision impairment (VI) places a burden on individuals, health systems and society in general. In order to support the case for investing in eye health services, an updated cost of illness study that measures the global impact of VI is necessary. To perform such a study, a systematic review of the literature is needed. Here we outline the protocol for a systematic review to describe and summarize the costs associated with VI and its major causes.

Methods and analysis

We will systematically search in MEDLINE (Ovid) and the CRD database (Centre for Reviews and Dissemination) which includes the National Health Service Economics Evaluation Database, the Database of Abstracts of Reviews of Effects and the Health Technology Assessment database. No language or geographical restriction will be applied. Additional literature will be identified by reviewing the references in included studies and by contacting field experts. Grey literature will be considered. The review will include any study published since 2000 that provides information about costs of illness, burden of disease and /or loss of well-being in participants with VI due to an unspecified cause or due to one of the seven leading causes globally.

Two reviewers will independently screen studies and extract relevant data from included studies. Methodological quality of economic studies will be assessed based on the British Medical Journal checklist for economic submissions adapted to costs of illness studies. This protocol has been prepared following the Preferred Reporting Items for Systematic review and meta-analysis protocols (PRISMA-P) and has been published prospectively in Open Science Framework.

Ethics and dissemination

Formal ethical approval is not required, as primary data will not be collected in this review. The findings of this study will be disseminated through peer-reviewed publications, stakeholder meetings, and inclusion in the ongoing Lancet Global Health Commission on Global Eye Health.

Registration details: https://osf.io/9au3w (DOI 10.17605/OSF.IO/6F8VM)

Keywords

Vision Impairment, Blindness, Cost of Illness, Ophthalmology

For peer review only

For peer review only - http://bmjopen.bmj.com/site/about/guidelines.xhtml

STRENGTHS AND LIMITATIONS OF THE STUDY

- ✓ This protocol adheres to the Preferred Reporting Items for Systematic review and meta-analysis protocols (PRISMA-P) and has been published prospectively in Open Science Framework

- ✓ This systematic review will search various databases extensively and will include studies published since 1 January 2000 without any language or geographical restriction

- ✓ All included studies will be appraised using the British Medical Journal Checklist for economic submissions adapted for cost of illness studies

- ✓ Due to the expected heterogeneity in study design, definitions of costs and loss of well- being, it is unlikely that a meta-analysis will be conducted.

For peer review only

BACKGROUND

Vision impairment (VI) is a major public health issue. In 2015 an estimated 36 million people (80% uncertainty interval 12.9 – 65.4) were blind (visual acuity worse than 3/60 in the better eye) and 216.6 million (80% uncertainty interval 98.5 – 359.1) were moderately or severely visually impaired (visual acuity better than 3/60 but worse than 6/18 in the better eye).[1] In 2015, 87% of blindness and 75% of moderate and severe vision impairment was due to seven causes—uncorrected refractive error, cataract, glaucoma, age-related macular degeneration (AMD), diabetic retinopathy, corneal opacity and trachoma.[2]

VI—being the combination of blindness and moderate and severe vision impairment—is associated with a range of consequences including difficulties performing activities of daily living,[3 4] reduced mobility,[5] higher risk of depression,[6] reduced educational outcomes,[7] impaired workplace productivity,[8] decreased quality of life,[9] increased risk of falls,[10] higher levels of dependency,[3] increased need for informal and formal care,[11 12] and an increased need for healthcare.[13 14] All of these lead to an economic burden for individuals, health systems and society. VI may occur at any age due to genetic, acquired or trauma related causes. However, prevalence increases with age in all world regions.

In 2010, the only global estimate of the cost of VI conducted to date was reported to be US$2954 billion,[15] with direct costs of US$2302 billion and informal care costs of US$246 million.[15] This analysis included productivity losses for high-income countries only, and in 2010 these were estimated to be US$168.3 billion.[15]

Another estimate of productivity losses due to VI has been reported in a study that used data from nine countries from high, middle and low-income countries and three different analysis approaches.[16] The most conservative of these approaches estimated that productivity losses due to VI in 2011 ranged from US$0.1 billion in Honduras to US$7.8 billion in the United States of America.[16] The authors concluded that although VI occurs more frequently in low and middle-income countries, the economic burden is still substantial in high-income countries like the United States of America and Japan.[16] Further, the full cost of VI is conceivably much higher if direct and informal care costs were included in estimates.

In order to make the case for investment and to develop plans to alleviate the burden of VI, an updated cost of illness study measuring the global impact from an economic and societal perspective is necessary.

For peer review only - http://bmjopen.bmj.com/site/about/guidelines.xhtml

Cost of illness studies measure the economic burden of a disease or condition on the overall population.[17 18] They are descriptive and analytic studies that estimate all direct health care costs, productivity and intangible costs of a disease or illness.[19] They are conducted to advise healthcare planners about the size of a problem in a population, to update and support policy and financing decisions and to inform full economic evaluation studies, namely cost-effectiveness and cost-benefit analyses.[20 21] Cost of illness studies do not compare alternative interventions and as such are considered partial economic evaluation studies.[22 23]

Cost of illness studies can be conducted from various perspectives, including societal, governmental, healthcare system, payer, healthcare provider and patient.[17] The analysis approach varies with the chosen perspective and may include direct medical costs, productivity costs, informal care costs and intangible costs.[21 24]

Cost of illness studies follow two different epidemiological approaches: prevalence or incidence-based approaches.[17 25] Prevalence-based studies estimate costs associated with prevalent cases over a given period of time, (usually 1 year), while incident-based studies estimate costs accrued over a lifetime following the onset of the illness or loss of health state.

Resource consumption estimates depend largely on the characteristics of the available data[17,24] and are usually categorized as top down ("population-level") or bottom up ("person-based").[17] Top down methods use aggregate expenditures by cost component while the bottom up method assigns costs to individuals with a specific disease or condition.

To perform a global cost of illness study, all available data must be identified and collated in a structured way. In 2012 a systematic review was conducted to inform a cost of illness study on VI and main causes of VI in high-income countries and a total of 22 studies were identified that reported direct and/or indirect costs related to VI.[26] Since 2012, new treatments (e.g. anti-vascular endothelial growth factor (anti-VEGF) therapy) and technologies (e.g. ocular imaging), have emerged. These are expected to increase direct costs and, if effective, improve outcomes.

A new systematic review is now required for three reasons. Firstly, the search will be extended to include low and middle as well as high-income countries to allow comprehensive global estimates. Secondly, we will expand the search to include the seven major causes of VI identified in the latest global prevalence estimates—cataract, uncorrected refractive error, diabetic retinopathy, glaucoma, AMD, corneal opacity and trachoma.[2] Finally, a new systematic review will capture studies on new treatments, such as anti-VEGF treatment, which

For peer review only - http://bmjopen.bmj.com/site/about/guidelines.xhtml

may result in both substantial costs and savings, and are thus likely to affect the societal cost of VI.

PURPOSE

The aim of this systematic review is to describe and summarize the costs associated with VI and its major causes.

METHODS AND ANALYSIS

The protocol is reported in accordance with the PRISMA-P Checklist[27 28] (Annex 2) and has been registered previously in Open Science Framework (https://osf.io/9au3w - doi10.17605/OSF.IO/6F8VM)).

Search

Literature searches will be performed in MEDLINE (Ovid) and the CRD database (Centre for Reviews and Dissemination) which includes the National Health Service Economics Evaluation Database (NHS EED), Database of Abstracts of Reviews of Effects (DARE) and the Health Technology Assessment (HTA) database. Searches will be run from the year 2000 onwards and no language or geographical restrictions will be applied. The search strategy is provided in Annex 1.

The reference lists of included articles will be reviewed for additional relevant articles. Field experts, including health economists and eye care researchers that have conducted economic evaluation in eye care, will be contacted to identify further potentially relevant studies and reports in the grey literature. These individuals will be identified from the authorship of the identified articles and snowballing via recommendations from Commissioners in the Lancet Commission on Global Eye Health.

Criteria

Studies will be included if they:

are partial economic evaluation studies such as cost of illness studies, burden of illness/diseases and full economic evaluation studies such as cost-effectiveness and cost-benefit studies published since 1 January 2000;

report in the results section a monetary estimate of the direct and/or indirect and/or productivity and/or informal care costs associated with persons with VI from an unspecified cause or due to one of the seven leading causes of vision loss globally (i.e.

For peer review only - http://bmjopen.bmj.com/site/about/guidelines.xhtml

cataract, uncorrected refractive error, diabetic retinopathy, glaucoma, AMD, corneal opacity and trachoma); and/or

report at least one of:

- undiscounted or discounted cost or benefit results; and/or
- an estimate of the impact of VI on labour market outcomes (e.g. employment chances, labour income, wages and lost work days), informal care (e.g. number of caregiver hours) or in terms of well-being (e.g. QALYS. DALYS).

Studies will be excluded if they:

only report incremental costs, net costs, incremental benefits or net benefits, incremental cost effectiveness ratio, incremental cost benefit ratios without also reporting actual costs; or

report costs and benefits related to specific eye diseases that are not one of the seven leading causes of vision loss globally; or

report costs of services for people with one of the major causes of VI (e.g. screening for everyone with diabetic retinopathy, providing medication for everyone with glaucoma) without specifically reporting the costs to deliver the service to people with VI. The exceptions will be studies reporting costs of services to treat cataract and refractive error—these will be included regardless of the vision status of participants, as they tend to be single (for cataract) or irregular (for refractive error) interventions that correct the VI, compared to the services required for the other causes; or

are reviews of existing economic studies related to VI; or

report an economic model based on other studies, but do not report any primary data.

Inclusion criteria are summarized and complemented with PICOS details in Table 1.

Table 1 – Summary of the PICOS elements for the systematic review

**Participants**	Participants with VI from an unspecified cause or due to one of the leading causes of VI globally (i.e. cataract, uncorrected refractive error, diabetic retinopathy, glaucoma, AMD, corneal opacity and trachoma)
**Interventions**	Any report that provides information about costs of illness, burden of diseases and /or loss of well-being in participants with VI or eye disease potentially leading to VI.

For peer review only - http://bmjopen.bmj.com/site/about/guidelines.xhtml

**Comparators**	Not relevant
**Outcomes**	Direct costs, indirect costs, productivity losses, informal care and intangible costs (e.g. Quality Adjusted Life Years, QALYs and Disability Adjusted Life Years, DALYs), transfer payments and deadweight losses.
**Study Design**	Partial economic evaluation studies such as cost of illness studies, burden of illness/diseases and full economic evaluation studies such as cost-effectiveness and cost-benefit studies

Cost classification description

Direct costs may include direct medical and non-medical costs associated with inpatient and outpatient care and all the resources used for diagnosis and treatment or eye disease and its sequels, long-term care and nursing home costs, community care and paid assistance provided by professionals, and costs related to vision aids and devices and home modifications) and transportation costs to access services.

Productivity costs (formerly called indirect costs) may include absenteeism, presenteeism, reduced workforce participation and loss productivity due to premature mortality.

Informal care may include hours spent by caregivers and/or a monetary estimate of the hours spent in care.

Intangible costs are captured through Quality Adjusted Life Years (QALYs), Disability Adjusted Life Years (DALYs).

Transfer payments such as social welfare payments made for distributional purposes.

Deadweight losses namely the cost to society of administering certain transfer payments, such as social welfare payments.

Selection of sources of evidence

All titles and abstracts will be screened by two investigators independently (APM and one of JR, JZ, TB) using Covidence systematic review software (Veritas Health Innovation, Melbourne, Australia. Available at www.covidence.org). After completing the screening process, full texts will be assessed by two investigators independently to establish eligibility for inclusion into the study. Since formal international guidelines for quality assessment of economic studies are lacking,[29] all included studies will be appraised by two investigators independently using the

For peer review only - http://bmjopen.bmj.com/site/about/guidelines.xhtml

British Medical Journal Checklist[30] for economic submissions adapted for cost of illness studies.[21] Any conflict in relation to screening and appraisal will be discussed between the two investigators, and resolved with a third investigator if necessary. A PRISMA flow diagram will be completed to summarise the study selection process.

Data extraction characteristics

The following information will be extracted from the included studies:

- Country or countries of study
- Study period
- Study size (e.g. population based studies or sampled based studies)
- Age range of participants
- Study design (e.g. cost of illness, burden of illness/diseases cost effectiveness or cost benefit studies)
- Epidemiological approach (e.g. incidence or prevalence based)
- Perspective of analysis (e.g. societal, government, healthcare system, payer, healthcare provider or patient)
- Main data sources (e.g. published expenditures report, administrative database, population survey, patient clinical records, patient diaries, specially designed questionnaires, published literature)
- Method of resource quantification (e.g. top-down or bottom-up)
- VI definition & VI severity (e.g blind, moderate or severe VI)
- Cause of VI (and definition) if specified
- Disease stage if specified
- Currency in which costs are reported
- Cost components (e.g. direct costs, productivity costs, informal care costs)
- Loss of well-being measures (e.g. intangible costs measured with QALYs, DALYs, years of sight loss)
- Analysis of uncertainty (e.g. type of uncertainty analysed (parameter uncertainty, methodological uncertainty or modelling uncertainty), choice of parameters included in sensitivity analysis, univariate sensitivity analysis, probabilistic sensitive analysis)
- Discounting methods (e.g. discount rate applied and justification)

If the study perspective or the epidemiological approach is not clearly specified in the studies, two investigators will assign a category for it by consensus.

Synthesis of results

Selected studies will be characterized in terms of country of origin, epidemiological approach, and perspective of analysis, study design, study size, methods of resource quantification and methods to deal with uncertainty.

We will describe the main reported cost categories, and the general assumptions and data sources used to estimate costs. Cost information will be summarised by VI severity level whenever this information is available. For better comparison across studies the reported

For peer review only - http://bmjopen.bmj.com/site/about/guidelines.xhtml

costs will be transformed by inflating to 2018 (or to the latest available year) using a country specific gross domestic product deflator and then converting to US dollar purchasing power parities (PPP, the rates of currency conversion that equalise the purchasing power of different currencies by accounting for differences in price levels between countries).

We will describe the main reported loss of well-being measures (QALYS, DALYS, etc), general assumptions and data sources. Loss of well-being will be summarized by VI severity level whenever this information is available.

Due to the expected heterogeneity in study design, definitions of costs / loss of well-being, it is unlikely that a meta-analysis will be conducted.

ETHICS AND DISSEMINATION

Formal ethical approval is not required, as primary data will not be collected in this review. The findings of this study will be disseminated through a peer-reviewed publication, stakeholder meetings, and inclusion in the ongoing Lancet Global Health Commission on Global Eye Health.[31]

PATIENT AND PUBLIC INVOLVEMENT

Patients and the public were not involved in the design of this systemic review protocol

For peer review only

For peer review only - http://bmjopen.bmj.com/site/about/guidelines.xhtml

REFERENCES

1. Bourne RRA, Flaxman SR, Braithwaite T, et al. Magnitude, temporal trends, and projections of the global prevalence of blindness and distance and near vision impairment: a systematic review and meta-analysis. *Lancet Glob Health* 2017;5(9):e888-e97. doi: 10.1016/s2214-109x(17)30293-0 [published Online First: 2017/08/07]
2. Flaxman SR, Bourne RRA, Resnikoff S, et al. Global causes of blindness and distance vision impairment 1990-2020: a systematic review and meta-analysis. *Lancet Glob Health* 2017;5(12):e1221-e34. doi: 10.1016/s2214-109x(17)30393-5 [published Online First: 2017/10/17]
3. West SK, Rubin GS, Broman AT, et al. How does visual impairment affect performance on tasks of everyday life? The SEE Project Salisbury Eye Evaluation. *Arch Ophthalmol* 2002;120(6):774-80.
4. Rubin GS, Munoz B, Bandeen-Roche K, et al. Monocular versus binocular visual acuity as measures of vision impairment and predictors of visual disability. *Invest Ophthalmol Vis Sci* 2000;41(11):3327-34. [published Online First: 2000/09/28]
5. Crews JE, Campbell VA. Vision impairment and hearing loss among community-dwelling older Americans: implications for health and functioning. *Am J Public Health* 2004;94(5):823-9. [published Online First: 2004/05/01]
6. Burmedi D, Becker S, Heyl V, et al. Emotional and social consequences of age-related low vision. *Visual Impairment Research* 2002;4(1):47-71. doi: 10.1076/vimr.4.1.47.15634
7. Ma X, Zhou Z, Yi H, et al. Effect of providing free glasses on children's educational outcomes in China: cluster randomized controlled trial. *Bmj* 2014;349:g5740. doi: 10.1136/bmj.g5740 [published Online First: 2014/09/25]
8. Reddy PA, Congdon N, MacKenzie G, et al. Effect of providing near glasses on productivity among rural Indian tea workers with presbyopia (PROSPER): a randomised trial. *The Lancet Global health* 2018;6(9):e1019-e27. doi: 10.1016/S2214-109X(18)30329-2
9. Brown MM, Brown GC, Sharma S, et al. Quality of life associated with visual loss: a time tradeoff utility analysis comparison with medical health states. *Ophthalmology* 2003;110(6):1076-81. doi: 10.1016/s0161-6420(03)00254-9 [published Online First: 2003/06/12]
10. Dhital A, Pey T, Stanford MR. Visual loss and falls: a review. *Eye (Lond)* 2010;24(9):1437-46. doi: 10.1038/eye.2010.60 [published Online First: 2010/05/08]
11. Hong T, Mitchell P, Burlutsky G, et al. Visual impairment and subsequent use of support services among older people: longitudinal findings from the Blue Mountains Eye Study. *Am J Ophthalmol* 2013;156(2):393-99 e1. doi: 10.1016/j.ajo.2013.04.002 [published Online First: 2013/05/22]
12. Wang JJ, Mitchell P, Cumming RG, et al. Visual impairment and nursing home placement in older Australians: the Blue Mountains Eye Study. *Ophthalmic Epidemiol* 2003;10(1):3-13. [published Online First: 2003/02/28]
13. Javitt JC, Zhou Z, Willke RJ. Association between vision loss and higher medical care costs in Medicare beneficiaries costs are greater for those with progressive vision loss. *Ophthalmology* 2007;114(2):238-45. doi: 10.1016/j.ophtha.2006.07.054 [published Online First: 2007/02/03]
14. Evans JR, Smeeth L, Fletcher AE. Hospital admissions in older people with visual impairment in Britain. *BMC Ophthalmol* 2008;8:16. doi: 10.1186/1471-2415-8-16 [published Online First: 2008/09/13]

For peer review only - http://bmjopen.bmj.com/site/about/guidelines.xhtml

15. Gordois A, Cutler H, Pezzullo L, et al. An estimation of the worldwide economic and health burden of visual impairment. *Glob Public Health* 2012;7(5):465-81. doi: 10.1080/17441692.2011.634815 [published Online First: 2011/12/06]
16. Eckert KA, Carter MJ, Lansingh VC, et al. A simple method for estimating the economic cost of productivity loss due to blindness and moderate to severe visual impairment. *Ophthalmic Epidemiol* 2015;22(5):349-55. doi: 10.3109/09286586.2015.1066394 [published Online First: 2015/09/24]
17. Larg A, Moss JR. Cost-of-illness studies: a guide to critical evaluation. *Pharmacoeconomics* 2011;29(8):653-71. doi: 10.2165/11588380-000000000-00000 [published Online First: 2011/05/25]
18. WHO. WHO guide to identifying the economic consequences of disease and injury. Geneve: World Health Organization, 2009.
19. Onukwugha E, McRae J, Kravetz A, et al. Cost-of-Illness Studies: An Updated Review of Current Methods. *Pharmacoeconomics* 2016;34(1):43-58. doi: 10.1007/s40273-015-0325-4 [published Online First: 2015/09/20]
20. Koopmanschap MA. Cost-of-illness studies. Useful for health policy? *Pharmacoeconomics* 1998;14(2):143-8. doi: 10.2165/00019053-199814020-00001 [published Online First: 1998/07/06]
21. Molinier L, Bauvin E, Combescure C, et al. Methodological considerations in cost of prostate cancer studies: a systematic review. *Value Health* 2008;11(5):878-85. doi: 10.1111/j.1524-4733.2008.00327.x [published Online First: 2008/05/23]
22. Drummond M, Sculpher M, Claxton K, et al. Methods for the economic evaluation of health care programmes. Oxford: Oxford University Press 2015.
23. Bloom BS, Bruno DJ, Maman DY, et al. Usefulness of US cost-of-illness studies in healthcare decision making. *Pharmacoeconomics* 2001;19(2):207-13. doi: 10.2165/00019053-200119020-00007 [published Online First: 2001/04/04]
24. Costa N, Derumeaux H, Rapp T, et al. Methodological considerations in cost of illness studies on Alzheimer disease. *Health Econ Rev* 2012;2(1):18. doi: 10.1186/2191-1991-2-18 [published Online First: 2012/09/12]
25. Lesyuk W, Kriza C, Kolominsky-Rabas P. Cost-of-illness studies in heart failure: a systematic review 2004-2016. *BMC Cardiovasc Disord* 2018;18(1):74. doi: 10.1186/s12872-018-0815-3 [published Online First: 2018/05/03]
26. Koberlein J, Beifus K, Schaffert C, et al. The economic burden of visual impairment and blindness: a systematic review. *BMJ Open* 2013;3(11):e003471. doi: 10.1136/bmjopen-2013-003471 [published Online First: 2013/11/10]
27. Shamseer L, Moher D, Clarke M, et al. Preferred reporting items for systematic review and meta-analysis protocols (PRISMA-P) 2015: elaboration and explanation. *BMJ* 2015;350:g7647. doi: 10.1136/bmj.g7647 [published Online First: 2015/01/04]
28. Moher D, Shamseer L, Clarke M, et al. Preferred reporting items for systematic review and meta-analysis protocols (PRISMA-P) 2015 statement. *Syst Rev* 2015;4:1. doi: 10.1186/2046-4053-4-1 [published Online First: 2015/01/03]
29. CRD. Systematic reviews:CRD´s guidance for undertaking reviews in health care. York: Centre for Reviews and Dissemination, University of York, 2009.
30. Drummond MF, Jefferson TO. Guidelines for authors and peer reviewers of economic submissions to the BMJ. The BMJ Economic Evaluation Working Party. *BMJ* 1996;313(7052):275-83. doi: 10.1136/bmj.313.7052.275 [published Online First: 1996/08/03]
31. Burton MJ, Faal HB, Ramke J, et al. Announcing The Lancet Global Health Commission on Global Eye Health. *Lancet Glob Health* 2019;7(12):e1612-e13. doi: 10.1016/s2214-109x(19)30450-4 [published Online First: 2019/10/14]

For peer review only - http://bmjopen.bmj.com/site/about/guidelines.xhtml

Authors´Contributions: APM, JC, JR and MJB conceived the idea for the review. APM and JR drafted and revised the protocol with suggestions from MJB, JC, TB, JHZ, HF, HT, IJ, NC, AB, TB, MJ, SR, RB, AN, PK and KF. IG constructed the search.

Funding Statement: MJB is supported by the Wellcome Trust (207472/Z/17/Z). JR is a Commonwealth Rutherford Fellow, funded by the UK government through the Commonwealth Scholarship Commission in the UK. The Lancet Global Health Commission on Global Eye Health is supported by The Queen Elizabeth Diamond Jubilee Trust, The Wellcome Trust, Sightsavers International, The Fred Hollows Foundation, The SEVA Foundation, The British Council for the Prevention of Blindness and Christian Blind Mission.

For peer review only

For peer review only - http://bmjopen.bmj.com/site/about/guidelines.xhtml

ANNEX 1 - SEARCH STRATEGIES

MEDLINE (Ovid)

1. "Global Burden of Disease"/
2. "costs and cost analysis"/
3. cost-benefit analysis/
4. "cost of illness"/
5. health care costs/
6. "Health Services Needs and Demand"/ec, sn [Economics, Statistics & Numerical Data]
7. Health Care Surveys/ec, sn [Economics, Statistics & Numerical Data]
8. Health Expenditures/ec, sn [Economics, Statistics & Numerical Data]
9. Health Resources/ec, sn [Economics, Statistics & Numerical Data]
10. Global Health/ec, sn [Economics, Statistics & Numerical Data]
11. ((global or economic) adj2 burden).tw.
12. ((cost or costs) adj2 (benefit or analysis or illness or direct or indirect or Intangible)).tw.
13. Efficiency/
14. Absenteeism/
15. Presenteeism/
16. productivity.tw.
17. "Severity of Illness Index"/ec [Economics]
18. Employment/ec [Economics]
19. Sick Leave/ec, sn [Economics, Statistics & Numerical Data]
20. (absenteeism or presenteeism or productivity).tw.
21. Caregivers/ec, sn [Economics, Statistics & Numerical Data]
22. or/1-21
23. exp eye diseases/
24. exp vision disorders/
25. ((vision or visual$) adj2 (impair$ or loss or disorder)).tw.
26. (cataract$ or glaucoma or macula$ degeneration).tw.
27. (diabetic retinopathy or refractive error$ or trachoma or corneal opacity).tw.
28. or/23-27
29. 22 and 28
30. limit 29 to yr="2000 -Current"

CRD database

The CRD database will be searched using the following MeSH terms:

MeSH DESCRIPTOR Eye Diseases EXPLODE ALL TREES

MeSH DESCRIPTOR Vision Disorders EXPLODE ALL TREES

For peer review only

ANNEX 2 - PRISMA-P 2015 CHECKLIST

This checklist has been adapted for use with protocol submissions to *Systematic Reviews* from Moher D et al: Preferred reporting items for systematic review and meta-analysis protocols (PRISMA-P) 2015 statement. *Systematic Reviews* **2015 4**:1

Section/topic	#	Checklist item	Information reported: Yes	Information reported: No	Line number(s)
**ADMINISTRATIVE INFORMATION**					
**Title**					
Identification	1a	Identify the report as a protocol of a systematic review	x		3 to 4 (page 1)
Update	1b	If the protocol is for an update of a previous systematic review, identify as such		x	
**Registration**	2	If registered, provide the name of the registry (e.g., PROSPERO) and registration number in the Abstract			55 (page 3)
**Authors**					
Contact	3a	Provide name, institutional affiliation, and e-mail address of all protocol authors; provide physical mailing address of corresponding author	X		10 to 59 (page 1)<br>3 to 13 (page 2)
Contributions	3b	Describe contributions of protocol authors and identify the guarantor of the review	X		3 to 7 (page 14)
**Amendments**	4	If the protocol represents an amendment of a previously completed or published protocol, identify as such and list changes; otherwise, state plan for documenting important protocol amendments		X	
**Support**					
Sources	5a	Indicate sources of financial or other support for the review	X		9 to 18 (page 14)
Sponsor	5b	Provide name for the review funder and/or sponsor		X	
Role of sponsor/funder	5c	Describe roles of funder(s), sponsor(s), and/or institution(s), if any, in developing the protocol		X	
**INTRODUCTION**					
**Rationale**	6	Describe the rationale for the review in the context of what is already known	X		6 to 57 (page 5)<br>3 to 59 (page 6)<br>3 to 4 (page 7)
**Objectives**	7	Provide an explicit statement of the question(s) the review will address with reference to participants, interventions, comparators, and outcomes (PICO)	X		10 to 12 (page 7)<br>44 to 60 (page 8)<br>2 to 16

Section/topic	#	Checklist item	Information reported: Yes	Information reported: No	Line number(s)
					(page 9)
**METHODS**					
**Eligibility criteria**	8	Specify the study characteristics (e.g., PICO, study design, setting, time frame) and report characteristics (e.g., years considered, language, publication status) to be used as criteria for eligibility for the review	X		46 to 58 (page 7) 3 to 60 (page 8) 2 to 16 (page 9)
**Information sources**	9	Describe all intended information sources (e.g., electronic databases, contact with study authors, trial registers, or other grey literature sources) with planned dates of coverage	X		26 to 45 (page 7)
**Search strategy**	10	Present draft of search strategy to be used for at least one electronic database, including planned limits, such that it could be repeated	X		3 to 49 Annex 1
STUDY RECORDS					
Data management	11a	Describe the mechanism(s) that will be used to manage records and data throughout the review	x		51 to 55 (page 9)
Selection process	11b	State the process that will be used for selecting studies (e.g., two independent reviewers) through each phase of the review (i.e., screening, eligibility, and inclusion in meta-analysis)	x		51 to 58 (page 9)
Data collection process	11c	Describe planned method of extracting data from reports (e.g., piloting forms, done independently, in duplicate), any processes for obtaining and confirming data from investigators	x		58 to 60 (page 9) 3 to 9 (page 10)
**Data items**	12	List and define all variables for which data will be sought (e.g., PICO items, funding sources), any pre-planned data assumptions and simplifications	x		12 to 43 (page 10)
**Outcomes and prioritization**	13	List and define all outcomes for which data will be sought, including prioritization of main and additional outcomes, with rationale	x		19 to 47 (page 9)
**Risk of bias in individual studies**	14	Describe anticipated methods for assessing risk of bias of individual studies, including whether this will be done at the outcome or study level, or both; state how this information will be used in data synthesis		X	
DATA					
**Synthesis**	15a	Describe criteria under which study data will be quantitatively synthesized		X	

For peer review only

Section/topic	#	Checklist item	Information reported Yes	No	Line number(s)
	15b	If data are appropriate for quantitative synthesis, describe planned summary measures, methods of handling data, and methods of combining data from studies, including any planned exploration of consistency (e.g., I^2, Kendall's tau)	☐	X☐	
	15c	Describe any proposed additional analyses (e.g., sensitivity or subgroup analyses, meta-regression)	☐	X☐	
	15d	If quantitative synthesis is not appropriate, describe the type of summary planned	X☐	☐	15 to 17 (page 11)
**Meta-bias(es)**	16	Specify any planned assessment of meta-bias(es) (e.g., publication bias across studies, selective reporting within studies)	☐	X☐	
**Confidence in cumulative evidence**	17	Describe how the strength of the body of evidence will be assessed (e.g., GRADE)	☐	X☐	58 to 60 (page 9)<br>3 to 5 (page 10)

For peer review only

BMJ Open

Estimating the global cost of Vision Impairment and its major causes: protocol for a systematic review

Journal:	*BMJ Open*
Manuscript ID	bmjopen-2019-036689.R1
Article Type:	Protocol
Date Submitted by the Author:	04-May-2020
Complete List of Authors:	Marques, Ana Patricia; London School of Hygiene and Tropical Medicine International Centre for Eye Health,<br>Ramke, Jacqueline; London School of Hygiene and Tropical Medicine, International Centre for Eye Health; University of Auckland, School of Optometry and Vision Science<br>Cairns, John; London School of Hygiene and Tropical Medicine, Department of Health Services Research and Policy<br>Butt, Thomas; University College London, UCL Institute of Ophthalmology<br>Zhang, Justine; London School of Hygiene and Tropical Medicine Faculty of Infectious and Tropical Diseases, International Centre for Eye Health, Clinical Research Department<br>Faal, Hannah; University of Calabar, Department of Ophthalmology<br>Taylor, Hugh; University of Melbourne School of Population and Global Health,<br>Jones, Iain; Sightsavers<br>Congdon, Nathan; Queen's University, Centre for Public Health; Sun Yat-Sen University Zhongshan Ophthalmic Center<br>Bastawrous, Andrew; London School of Hygiene and Tropical Medicine, International Centre for Eye Health<br>Braithwaite, Tasanee; London School of Hygiene and Tropical Medicine, International Centre for Eye Health; Moorfields Eye Hospital<br>Jovic, Marty; PricewaterhouseCoopers<br>Resnikoff, Serge; University of New South Wales, Brien Holden Vision Institute<br>Nandakumar, Allayala; Brandeis University Heller School for Social Policy and Management<br>Khaw, Peng; NIHR Biomedical Research Centre at Moorfields Eye Hospital and UCL Institute of Ophthalmology<br>Bourne, Rupert; Anglia Ruskin University, Vision and Eye Research Unit<br>Gordon, Iris; London School of Hygiene and Tropical Medicine, International Centre for Eye Health<br>Frick, Kevin; Johns Hopkins University Carey Business School - Baltimore Campus<br>Burton, Matthew J; London School of Hygiene and Tropical Medicine, International Centre for Eye Health; Moorfields Eye Hospital
**Primary Subject Heading**:	Ophthalmology
Secondary Subject Heading:	Health economics

Keywords:	OPHTHALMOLOGY, PUBLIC HEALTH, HEALTH ECONOMICS

SCHOLARONE™
Manuscripts

I, the Submitting Author has the right to grant and does grant on behalf of all authors of the Work (as defined in the below author licence), an exclusive licence and/or a non-exclusive licence for contributions from authors who are: i) UK Crown employees; ii) where BMJ has agreed a CC-BY licence shall apply, and/or iii) in accordance with the terms applicable for US Federal Government officers or employees acting as part of their official duties; on a worldwide, perpetual, irrevocable, royalty-free basis to BMJ Publishing Group Ltd ("BMJ") its licensees and where the relevant Journal is co-owned by BMJ to the co-owners of the Journal, to publish the Work in this journal and any other BMJ products and to exploit all rights, as set out in our licence.

The Submitting Author accepts and understands that any supply made under these terms is made by BMJ to the Submitting Author unless you are acting as an employee on behalf of your employer or a postgraduate student of an affiliated institution which is paying any applicable article publishing charge ("APC") for Open Access articles. Where the Submitting Author wishes to make the Work available on an Open Access basis (and intends to pay the relevant APC), the terms of reuse of such Open Access shall be governed by a Creative Commons licence – details of these licences and which Creative Commons licence will apply to this Work are set out in our licence referred to above.

Other than as permitted in any relevant BMJ Author's Self Archiving Policies, I confirm this Work has not been accepted for publication elsewhere, is not being considered for publication elsewhere and does not duplicate material already published. I confirm all authors consent to publication of this Work and authorise the granting of this licence.

For peer review only

ESTIMATING THE GLOBAL COST OF VISION IMPAIRMENT AND ITS MAJOR CAUSES: PROTOCOL FOR A SYSTEMATIC REVIEW

Authors:

Ana Patricia Marques[1], Jacqueline Ramke[1,2], John Cairns[3], Thomas Butt[4] , Justine H. Zhang[1,5], Hannah Faal[6], Hugh Taylor[7], Iain Jones[8], Nathan Congdon[9,10], Andrew Bastawrous[1], Tasanee Braithwaite[1,11], Marty Jovic[12], Serge Resnikoff[13], Allayala Nandakumar[14], Peng T. Khaw[15], Rupert Bourne[16], Iris Gordon[1], Kevin Frick[17], Matthew J. Burton[1,11]

Affiliations:

1. International Centre for Eye Health, London School of Hygiene and Tropical Medicine, London, United Kingdom
2. School of Optometry and Vision Science, University of Auckland, Auckland, New Zealand
3. Health Services Research and Policy, London School of Hygiene and Tropical Medicine, London, United Kingdom
4. UCL Institute of Ophthalmology, University College London, London, United Kingdom
5. Manchester Royal Eye Hospital, Manchester, United Kingdom
6. Department of Ophthalmology, University of Calabar, Calabar, Nigeria
7. Indigenous Eye Health Unit, Melbourne School of Population and Global Health, The University of Melbourne, Melbourne, Australia
8. Sightsavers, Haywards Heath, United Kingdom
9. Centre for Public Health, Queen's University, Belfast, United Kingdom
10. Zhongshan Ophthalmic Center, Sun Yat-sen University, Guangzhou, China
11. Moorfields Eye Hospital, London, United Kingdom
12. PricewaterhouseCoopers, Sydney, Australia
13. Brien Holden Vision Institute and SOVS, University of New South Wales, Sydney, NSW, Australia
14. Heller School for Social Policy and Management, Brandeis University, Waltham, Massachusetts, United States of America
15. National Institute for Health Research Biomedical Research Centre, Moorfields Eye Hospital NHS Foundation Trust and UCL Institute of Ophthalmology, London, United Kingdom
16. Vision and Eye Research Institute, School of Medicine, Anglia Ruskin University, Cambridge, United Kingdom
17. Johns Hopkins Carey Business School, Baltimore, Maryland, United States of America

For peer review only - http://bmjopen.bmj.com/site/about/guidelines.xhtml

Corresponding author: Ana Patricia Marques

Address: International Centre for Eye Health, London School of Hygiene and Tropical Medicine, Keppel Street, London, WC1E 7HT

E-mail: Patricia.Marques@lshtm.ac.uk

E-mail addresses of all co-authors are available from the corresponding author upon reasonable request.

Word Count: ~2600

For peer review only

For peer review only - http://bmjopen.bmj.com/site/about/guidelines.xhtml

ABSTRACT

Introduction

Vision impairment (VI) places a burden on individuals, health systems and society in general. In order to support the case for investing in eye health services, an updated cost of illness study that measures the global impact of VI is necessary. To perform such a study, a systematic review of the literature is needed. Here we outline the protocol for a systematic review to describe and summarize the costs associated with VI and its major causes.

Methods and analysis

We will systematically search in MEDLINE (Ovid) and the CRD database (Centre for Reviews and Dissemination) which includes the National Health Service Economics Evaluation Database. No language or geographical restriction will be applied. Additional literature will be identified by reviewing the references in included studies and by contacting field experts. Grey literature will be considered. The review will include any study published from 1 January 2000 to November 2019 that provides information about costs of illness, burden of disease and /or loss of well-being in participants with VI due to an unspecified cause or due to one of the seven leading causes globally.

Two reviewers will independently screen studies and extract relevant data from included studies. Methodological quality of economic studies will be assessed based on the British Medical Journal checklist for economic submissions adapted to costs of illness studies. This protocol has been prepared following the Preferred Reporting Items for Systematic review and meta-analysis protocols (PRISMA-P) and has been published prospectively in Open Science Framework.

Ethics and dissemination

Formal ethical approval is not required, as primary data will not be collected in this review. The findings of this study will be disseminated through peer-reviewed publications, stakeholder meetings, and inclusion in the ongoing Lancet Global Health Commission on Global Eye Health.

Registration details: https://osf.io/9au3w (DOI 10.17605/OSF.IO/6F8VM)

Keywords

Vision Impairment, Blindness, Cost of Illness, Ophthalmology

For peer review only - http://bmjopen.bmj.com/site/about/guidelines.xhtml

STRENGTHS AND LIMITATIONS OF THE STUDY

- ✓ This protocol adheres to the Preferred Reporting Items for Systematic review and meta-analysis protocols (PRISMA-P) and has been published prospectively in Open Science Framework
- ✓ This systematic review will search various databases extensively and will include studies published since 1 January 2000 to November 2019 without any language or geographical restriction
- ✓ All included studies will be appraised using the British Medical Journal Checklist for economic submissions adapted for cost of illness studies
- ✓ Synthesis of findings will be difficult as resource use (including diagnostic procedures and treatment options) and costs will likely vary between countries, over time and according to which cause(s) of vision loss is reported – in lieu of synthesis we will summarise the range and quality of available evidence, and the subsequent gaps where evidence should be produced and improved
- ✓ Due to the expected heterogeneity in study methods it is unlikely that a meta-analysis will be conducted

For peer review only

For peer review only - http://bmjopen.bmj.com/site/about/guidelines.xhtml

BACKGROUND

Vision impairment (VI) is a major public health issue. In 2015 an estimated 36 million people (80% uncertainty interval 12.9 – 65.4) were blind (visual acuity worse than 3/60 in the better eye) and 216.6 million (80% uncertainty interval 98.5 – 359.1) were moderately or severely visually impaired (visual acuity better than 3/60 but worse than 6/18 in the better eye).[1] In 2015, 87% of blindness and 75% of moderate and severe vision impairment was due to seven causes—uncorrected refractive error, cataract, glaucoma, age-related macular degeneration (AMD), diabetic retinopathy, corneal opacity and trachoma.[2]

VI—being the combination of blindness and moderate and severe vision impairment—is associated with a range of consequences including difficulties performing activities of daily living,[3-5] reduced mobility,[6] higher risk of depression,[7 8] reduced educational outcomes,[9] impaired workplace productivity,[10] decreased quality of life,[11] increased risk of falls,[12] higher levels of dependency,[3] increased need for informal and formal care,[13-15] and an increased need for healthcare.[16-18] All of these lead to an economic burden for individuals, health systems and society. VI may occur at any age due to genetic, acquired or trauma related causes. However, prevalence increases with age in all world regions.

In 2010, the only global estimate of the cost of VI conducted to date was reported to be US$2954 billion,[19] with direct costs of US$2302 billion and informal care costs of US$246 million.[19] This analysis included productivity losses for high-income countries only, and in 2010 these were estimated to be US$168.3 billion.[19]

Another estimate of productivity losses due to VI has been reported in a study that used data from nine countries from high, middle and low-income countries and three different analysis approaches.[20] The most conservative of these approaches estimated that productivity losses due to VI in 2011 ranged from US$0.1 billion in Honduras to US$7.8 billion in the United States of America.[20] The authors concluded that although VI occurs more frequently in low and middle-income countries, the economic burden is still substantial in high-income countries like the United States of America and Japan.[20] Further, the full cost of VI is conceivably much higher if direct and informal care costs were included in estimates.

In order to make the case for investment and to develop plans to alleviate the burden of VI, an updated cost of illness study measuring the global impact from an economic and societal perspective is necessary.

For peer review only - http://bmjopen.bmj.com/site/about/guidelines.xhtml

Cost of illness studies measure the economic burden of a disease or condition on the overall population.[21 22] They are descriptive and analytic studies that estimate all direct health care costs, productivity and intangible costs of a disease or illness.[23] They are conducted to advise healthcare planners about the size of a problem in a population, to update and support policy and financing decisions and to inform full economic evaluation studies, namely cost-effectiveness and cost-benefit analyses.[24 25] Cost of illness studies do not compare alternative interventions and as such are considered partial economic evaluation studies.[26 27]

To perform a global cost of illness study, all available data must be identified and collated in a structured way. In 2012 a systematic review was conducted to inform a cost of illness study on VI and main causes of VI in high-income countries and a total of 22 studies were identified that reported direct and/or indirect costs related to VI.[28] Since 2012, new treatments (e.g. anti-vascular endothelial growth factor (anti-VEGF) therapy) and technologies (e.g. ocular imaging), have emerged. These are expected to increase direct costs and, if effective, improve outcomes.

A new systematic review is now required for three reasons. Firstly, the search will be extended to include low and middle as well as high-income countries to allow comprehensive global estimates. Secondly, we will expand the search to include the seven major causes of VI identified in the latest global prevalence estimates—cataract, uncorrected refractive error, diabetic retinopathy, glaucoma, AMD, corneal opacity and trachoma.[2] Finally, a new systematic review will capture studies on new treatments, such as anti-VEGF treatment, which may result in both substantial costs and savings, and are thus likely to affect the societal cost of VI.

PURPOSE

The aim of this systematic review is to describe and summarize the costs associated with VI and its major causes.

METHODS AND ANALYSIS

The protocol is reported in accordance with the PRISMA-P Checklist[29 30] (Annex 1) and has been registered previously in Open Science Framework (https://osf.io/9au3w - doi10.17605/OSF.IO/6F8VM)).

For peer review only - http://bmjopen.bmj.com/site/about/guidelines.xhtml

Search

Literature searches will be performed in MEDLINE (Ovid) and the CRD database (Centre for Reviews and Dissemination) which includes the National Health Service Economics Evaluation Database (NHS EED), Database of Abstracts of Reviews of Effects (DARE) and the Health Technology Assessment (HTA) database. Searches will be run to identify studies published from 1 January 2000 to November 2019 and no language or geographical restrictions will be applied. The search strategy is provided in Annex 2.

The reference lists of included articles will be reviewed for additional relevant articles. Field experts, including health economists and eye care researchers that have conducted economic evaluation in eye care, will be contacted to identify further potentially relevant studies and reports in the grey literature. These individuals will be identified from the authorship of the identified articles and snowballing via recommendations from Commissioners in the Lancet Commission on Global Eye Health.

Criteria

Studies will be included if they:

- are partial economic evaluation studies such as cost of illness studies, burden of illness/diseases and full economic evaluation studies such as cost-effectiveness and cost-benefit studies published since 1 January 2000;
- report in the results section a monetary estimate of the direct and/or indirect and/or productivity and/or informal care costs associated with persons with VI from an unspecified cause or due to one of the seven leading causes of vision loss globally (i.e. cataract, uncorrected refractive error, diabetic retinopathy, glaucoma, AMD, corneal opacity and trachoma); and/or
- report at least one of:
  - undiscounted or discounted cost or benefit results; and/or
  - an estimate of the impact of VI on labour market outcomes (e.g. employment chances, labour income, wages and lost work days), informal care (e.g. number of caregiver hours) or in terms of well-being (e.g. Quality Adjusted Life Years (QALYs), Disability Adjusted Life Years (DALYs).
  - Studies will be excluded if they:
- only report incremental costs, net costs, incremental benefits or net benefits, incremental cost effectiveness ratio, incremental cost benefit ratios without also reporting actual costs; or

For peer review only - http://bmjopen.bmj.com/site/about/guidelines.xhtml

- report costs and benefits related to specific eye diseases that are not one of the seven leading causes of vision loss globally; or
- report costs of services for people with one of the major causes of VI (e.g. screening for everyone with diabetic retinopathy, providing medication for everyone with glaucoma) without specifically reporting the costs to deliver the service to people with VI. The exceptions will be studies reporting costs of services to treat cataract and refractive error—these will be included regardless of the vision status of participants, as they tend to be single (for cataract) or irregular (for refractive error) interventions that correct the VI, compared to the services required for the other causes; or
- are reviews of existing economic studies related to VI; or
- report an economic model based on other studies, but do not report any primary costs data.

Inclusion criteria are summarized and complemented with PICOS details in Table 1.

Table 1 – Summary of the PICOS elements for the systematic review

**P**articipants	Participants with VI from an unspecified cause or due to one of the leading causes of VI globally (i.e. cataract, uncorrected refractive error, diabetic retinopathy, glaucoma, AMD, corneal opacity and trachoma)
**I**nterventions	Any report that provides information about costs of illness, burden of diseases and /or loss of well-being in participants with VI or eye disease potentially leading to VI.
**C**omparators	Not relevant
**O**utcomes	Direct costs, indirect costs, productivity losses, informal care and intangible costs (e.g. Quality Adjusted Life Years, QALYs and Disability Adjusted Life Years, DALYs), transfer payments and deadweight losses.
**S**tudy Design	Partial economic evaluation studies such as cost of illness studies, burden of illness/diseases and full economic evaluation studies such as cost-effectiveness and cost-benefit studies. Model based economic evaluation studies not reporting any primary cost data or based on reviews of existing economic studies were excluded.

For peer review only - http://bmjopen.bmj.com/site/about/guidelines.xhtml

Methodological features of cost of illness studies

Cost of illness studies follow two different epidemiological approaches: prevalence or incidence-based approaches.[21 31] Prevalence-based studies estimate costs associated with prevalent cases over a given period of time, (usually 1 year), while incident-based studies estimate costs accrued over a lifetime following the onset of the illness or loss of health state.

Cost of illness studies can be conducted from various perspectives, including societal, governmental, healthcare system, payer, healthcare provider and patient.[21] The analysis approach varies with the chosen perspective and may include direct costs, productivity costs, informal care costs and intangible costs.[25 32]

Direct costs may include direct medical and non-medical costs associated with inpatient and outpatient care and all the resources used for diagnosis and treatment or eye disease and its sequelae, long-term care and nursing home costs, community care and paid assistance provided by professionals, costs related to vision aids and devices and home modifications and transportation costs to access services. **Productivity costs** (formerly called indirect costs) may include absenteeism, presenteeism, reduced workforce participation and loss productivity due to premature mortality**.** **Informal care** may include hours spent by caregivers and/or a monetary estimate of the hours spent in care. **Intangible costs** are captured through QALYs and DALYs. **Transfer payments** such as social welfare payments made for distributional purposes. **Deadweight losses** namely the cost to society of administering certain transfer payments, such as social welfare payments.

Resource consumption estimates depend largely on the characteristics of the available data[21,32] and are usually categorized as top down ("population-level") or bottom up ("person-based").[21] Top down methods use aggregate expenditures by cost component while the bottom up method assigns costs to individuals with a specific disease or condition.

Selection of sources of evidence

All titles and abstracts will be screened by two investigators independently (APM and one of JR, JZ, TB) using Covidence systematic review software (Veritas Health Innovation, Melbourne, Australia. Available at www.covidence.org). After completing the screening process, full texts will be assessed by two investigators independently to establish eligibility for inclusion into the

For peer review only - http://bmjopen.bmj.com/site/about/guidelines.xhtml

study. Since formal international guidelines for quality assessment of economic studies are lacking,[33] all included studies will be appraised by two investigators independently using the British Medical Journal Checklist[34] for economic submissions adapted for cost of illness studies.[25] Each quality criteria will be scored as one of "yes," "no," "partial," or "not applicable". We will follow the approach used several times previously to identify the methodological strengths and weakness of the included studies [32 35 36] – equal weight will be assigned to each item of the checklist and the final score will be equal to the sum of the 10 individual items. Any conflict in relation to screening and appraisal will be discussed between the two investigators, and resolved with a third investigator if necessary. A PRISMA flow diagram will be completed to summarise the study selection process.

Data extraction characteristics

The following information will be extracted from the included studies:

- Country or countries of study
- Study period
- Study size (e.g. population based studies or sampled based studies)
- Age range of participants
- Study design (e.g. cost of illness, burden of illness/diseases cost effectiveness or cost benefit studies)
- Epidemiological approach (e.g. incidence or prevalence based)
- Perspective of analysis (e.g. societal, government, healthcare system, payer, healthcare provider or patient)
- Main data sources (e.g. published expenditures report, administrative database, population survey, patient clinical records, patient diaries, specially designed questionnaires, published literature)
- Method of resource quantification (e.g. top-down or bottom-up)
- VI definition & VI severity (e.g blind, moderate or severe VI)
- Cause of VI (and definition)
- Disease stage
- Currency in which costs are reported
- Cost components (e.g. direct costs, productivity costs, informal care costs)
- Loss of well-being measures (e.g. intangible costs measured with QALYs, DALYs, years of sight loss)
- Analysis of uncertainty (e.g. type of uncertainty analysed (parameter uncertainty, methodological uncertainty or modelling uncertainty), choice of parameters included in sensitivity analysis, univariate sensitivity analysis, probabilistic sensitive analysis)
- Discounting methods (e.g. discount rate applied and justification)

If the study perspective or the epidemiological approach is not clearly specified in the studies, two investigators will assign a category for it by consensus.

For peer review only - http://bmjopen.bmj.com/site/about/guidelines.xhtml

Synthesis of results

Selected studies will be characterized in terms of country of origin, epidemiological approach, perspective of analysis, study design, study size, methods of resource quantification and methods to deal with uncertainty.

We will describe the main reported cost categories and the general assumptions used to estimate costs. We will take four steps to prepare study results for comparison:

1) we will categorise studies either as 'general' studies that reported costs for people with blindness or VI or 'condition' specific studies that reported costs for people with one of the seven specified causes of vision loss;

2) if costs per patient per year are not reported for national or global estimates studies, these will be calculated for studies where sufficient information is provided;

3) costs will be inflated to 2018 values (or to the recent available year) using country specific Gross Domestic Product (GDP) deflators[37]; and

4) costs will be converted to USD purchasing power parities (PPP)[38] to equalise the purchasing power of different currencies.

Time transformations will adjust for inflation costs reported in the same country but in different years. Conversion to USD PPP conversion will adjust for the same price level costs estimates reported in different countries and different currencies. This cost transformation will convert all reported costs to the same year (2018), same currency and same purchasing power (USD PPP).

Due to anticipated heterogeneity in the cost data, studies will be stratified and presented by the four different costs components (i.e Direct Costs, Productivity losses, Informal care and Intangible costs), with a clear explanation of what has been included in each of the four cost components. A table summarizing which items are included in the four major costs components will be reported to summarise the similarities and differences between studies. Costs data will also be stratified by severity of VI when this information is available. Since this systematic review aims to collect data to assist a future global economic estimate for VI and its major causes, the transformed costs per patient per year stratified by costs components will be aggregated by GDB Regions and GBD Super Regions. Descriptive measures will be calculated to report the costs per patient per year for each GDB region and super region (e.g mean, standard deviation, minimum and maximum).

For peer review only - http://bmjopen.bmj.com/site/about/guidelines.xhtml

We will describe the main reported loss of well-being measures and its general assumptions. Loss of well-being measures will be summarized in their natural units (e.g. QALYS and DALYS) rather than reported in their monetized value since there is no consensus on assigning a monetary value to health outcomes [21 26 39] and because there is no common acceptable value across countries.

Due to the expected heterogeneity in study design, definitions of costs / loss of well-being [40], it is unlikely that a meta-analysis will be conducted.

ETHICS AND DISSEMINATION

Formal ethical approval is not required, as primary data will not be collected in this review. The findings of this study will be disseminated through a peer-reviewed publication, stakeholder meetings, and inclusion in the ongoing Lancet Global Health Commission on Global Eye Health.[41]

PATIENT AND PUBLIC INVOLVEMENT

Patients and the public were not involved in the design of this systemic review protocol.

For peer review only

For peer review only - http://bmjopen.bmj.com/site/about/guidelines.xhtml

REFERENCES

1. Bourne RRA, Flaxman SR, Braithwaite T, et al. Magnitude, temporal trends, and projections of the global prevalence of blindness and distance and near vision impairment: a systematic review and meta-analysis. *Lancet Glob Health* 2017;5(9):e888-e97. doi: 10.1016/s2214-109x(17)30293-0 [published Online First: 2017/08/07]
2. Flaxman SR, Bourne RRA, Resnikoff S, et al. Global causes of blindness and distance vision impairment 1990-2020: a systematic review and meta-analysis. *Lancet Glob Health* 2017;5(12):e1221-e34. doi: 10.1016/s2214-109x(17)30393-5 [published Online First: 2017/10/17]
3. West SK, Rubin GS, Broman AT, et al. How does visual impairment affect performance on tasks of everyday life? The SEE Project Salisbury Eye Evaluation. *Arch Ophthalmol* 2002;120(6):774-80.
4. Rubin GS, Munoz B, Bandeen-Roche K, et al. Monocular versus binocular visual acuity as measures of vision impairment and predictors of visual disability. *Invest Ophthalmol Vis Sci* 2000;41(11):3327-34. [published Online First: 2000/09/28]
5. Black AA, Wood JM, Lovie-Kitchin JE. Inferior visual field reductions are associated with poorer functional status among older adults with glaucoma. *Ophthalmic Physiol Opt* 2011;31(3):283-91. doi: 10.1111/j.1475-1313.2010.00811.x [published Online First: 2011/03/18]
6. Crews JE, Campbell VA. Vision impairment and hearing loss among community-dwelling older Americans: implications for health and functioning. *Am J Public Health* 2004;94(5):823-9. [published Online First: 2004/05/01]
7. Burmedi D, Becker S, Heyl V, et al. Emotional and social consequences of age-related low vision. *Visual Impairment Research* 2002;4(1):47-71. doi: 10.1076/vimr.4.1.47.15634
8. Zheng Y, Wu X, Lin X, et al. The Prevalence of Depression and Depressive Symptoms among Eye Disease Patients: A Systematic Review and Meta-analysis. *Sci Rep* 2017;7:46453. doi: 10.1038/srep46453 [published Online First: 2017/04/13]
9. Ma X, Zhou Z, Yi H, et al. Effect of providing free glasses on children's educational outcomes in China: cluster randomized controlled trial. *Bmj* 2014;349:g5740. doi: 10.1136/bmj.g5740 [published Online First: 2014/09/25]
10. Reddy PA, Congdon N, MacKenzie G, et al. Effect of providing near glasses on productivity among rural Indian tea workers with presbyopia (PROSPER): a randomised trial. *The Lancet Global health* 2018;6(9):e1019-e27. doi: https://dx.doi.org/10.1016/S2214-109X(18)30329-2
11. Brown MM, Brown GC, Sharma S, et al. Quality of life associated with visual loss: a time tradeoff utility analysis comparison with medical health states. *Ophthalmology* 2003;110(6):1076-81. doi: 10.1016/s0161-6420(03)00254-9 [published Online First: 2003/06/12]
12. Dhital A, Pey T, Stanford MR. Visual loss and falls: a review. *Eye (Lond)* 2010;24(9):1437-46. doi: 10.1038/eye.2010.60 [published Online First: 2010/05/08]
13. Hong T, Mitchell P, Burlutsky G, et al. Visual impairment and subsequent use of support services among older people: longitudinal findings from the Blue Mountains Eye Study. *Am J Ophthalmol* 2013;156(2):393-99 e1. doi: 10.1016/j.ajo.2013.04.002 [published Online First: 2013/05/22]
14. Wang JJ, Mitchell P, Cumming RG, et al. Visual impairment and nursing home placement in older Australians: the Blue Mountains Eye Study. *Ophthalmic Epidemiol* 2003;10(1):3-13. [published Online First: 2003/02/28]
15. Marques AP, Macedo AF, Hernandez-Moreno L, et al. The use of informal care by people with vision impairment. *PloS one* 2018;13(6):e0198631. doi: https://dx.doi.org/10.1371/journal.pone.0198631

For peer review only - http://bmjopen.bmj.com/site/about/guidelines.xhtml

16. Javitt JC, Zhou Z, Willke RJ. Association between vision loss and higher medical care costs in Medicare beneficiaries costs are greater for those with progressive vision loss. *Ophthalmology* 2007;114(2):238-45. doi: 10.1016/j.ophtha.2006.07.054 [published Online First: 2007/02/03]
17. Evans JR, Smeeth L, Fletcher AE. Hospital admissions in older people with visual impairment in Britain. *BMC Ophthalmol* 2008;8:16. doi: 10.1186/1471-2415-8-16 [published Online First: 2008/09/13]
18. Wang M-T, Ng K, Sheu S-J, et al. Analysis of Excess Direct Medical Costs of Vision Impairment in Taiwan. *Value in health regional issues* 2013;2(1):57-63. doi: https://dx.doi.org/10.1016/j.vhri.2013.01.006
19. Gordois A, Cutler H, Pezzullo L, et al. An estimation of the worldwide economic and health burden of visual impairment. *Glob Public Health* 2012;7(5):465-81. doi: 10.1080/17441692.2011.634815 [published Online First: 2011/12/06]
20. Eckert KA, Carter MJ, Lansingh VC, et al. A simple method for estimating the economic cost of productivity loss due to blindness and moderate to severe visual impairment. *Ophthalmic Epidemiol* 2015;22(5):349-55. doi: 10.3109/09286586.2015.1066394 [published Online First: 2015/09/24]
21. Larg A, Moss JR. Cost-of-illness studies: a guide to critical evaluation. *Pharmacoeconomics* 2011;29(8):653-71. doi: 10.2165/11588380-000000000-00000 [published Online First: 2011/05/25]
22. WHO. WHO guide to identifying the economic consequences of disease and injury. Geneve: World Health Organization, 2009.
23. Onukwugha E, McRae J, Kravetz A, et al. Cost-of-Illness Studies: An Updated Review of Current Methods. *Pharmacoeconomics* 2016;34(1):43-58. doi: 10.1007/s40273-015-0325-4 [published Online First: 2015/09/20]
24. Koopmanschap MA. Cost-of-illness studies. Useful for health policy? *Pharmacoeconomics* 1998;14(2):143-8. doi: 10.2165/00019053-199814020-00001 [published Online First: 1998/07/06]
25. Molinier L, Bauvin E, Combescure C, et al. Methodological considerations in cost of prostate cancer studies: a systematic review. *Value Health* 2008;11(5):878-85. doi: 10.1111/j.1524-4733.2008.00327.x [published Online First: 2008/05/23]
26. Drummond M, Sculpher M, Claxton K, et al. Methods for the economic evaluation of health care programmes. Oxford: Oxford University Press 2015.
27. Bloom BS, Bruno DJ, Maman DY, et al. Usefulness of US cost-of-illness studies in healthcare decision making. *Pharmacoeconomics* 2001;19(2):207-13. doi: 10.2165/00019053-200119020-00007 [published Online First: 2001/04/04]
28. Koberlein J, Beifus K, Schaffert C, et al. The economic burden of visual impairment and blindness: a systematic review. *BMJ Open* 2013;3(11):e003471. doi: 10.1136/bmjopen-2013-003471 [published Online First: 2013/11/10]
29. Shamseer L, Moher D, Clarke M, et al. Preferred reporting items for systematic review and meta-analysis protocols (PRISMA-P) 2015: elaboration and explanation. *BMJ* 2015;350:g7647. doi: 10.1136/bmj.g7647 [published Online First: 2015/01/04]
30. Moher D, Shamseer L, Clarke M, et al. Preferred reporting items for systematic review and meta-analysis protocols (PRISMA-P) 2015 statement. *Syst Rev* 2015;4:1. doi: 10.1186/2046-4053-4-1 [published Online First: 2015/01/03]
31. Lesyuk W, Kriza C, Kolominsky-Rabas P. Cost-of-illness studies in heart failure: a systematic review 2004-2016. *BMC Cardiovasc Disord* 2018;18(1):74. doi: 10.1186/s12872-018-0815-3 [published Online First: 2018/05/03]
32. Costa N, Derumeaux H, Rapp T, et al. Methodological considerations in cost of illness studies on Alzheimer disease. *Health Econ Rev* 2012;2(1):18. doi: 10.1186/2191-1991-2-18 [published Online First: 2012/09/12]

For peer review only - http://bmjopen.bmj.com/site/about/guidelines.xhtml

33. CRD. Systematic reviews:CRD´s guidance for undertaking reviews in health care. York: Centre for Reviews and Dissemination, University of York, 2009.
34. Drummond MF, Jefferson TO. Guidelines for authors and peer reviewers of economic submissions to the BMJ. The BMJ Economic Evaluation Working Party. *BMJ* 1996;313(7052):275-83. doi: 10.1136/bmj.313.7052.275 [published Online First: 1996/08/03]
35. Wang L, Si L, Cocker F, et al. A Systematic Review of Cost-of-Illness Studies of Multimorbidity. *Appl Health Econ Health Policy* 2018;16(1):15-29. doi: 10.1007/s40258-017-0346-6 [published Online First: 2017/09/01]
36. Shafie AA, Tan YP, Ng CH. Systematic review of economic burden of heart failure. *Heart Fail Rev* 2018;23(1):131-45. doi: 10.1007/s10741-017-9661-0 [published Online First: 2017/11/11]
37. World Bank. World Development Indicators: GDP deflator (base year varies by country). Washington: The World Bank Group, 2020.
38. World Bank. World Development Indicators: PPP conversion factor, GDP (LCU per international $). Washington: World Bank, 2020.
39. Frick KD, Gower EW, Kempen JH, et al. Economic impact of visual impairment and blindness in the United States. *Archives of ophthalmology (Chicago, Ill : 1960)* 2007;125(4):544-50.
40. Chisholm D, Stanciole AE, Tan Torres Edejer T, et al. Economic impact of disease and injury: counting what matters. *Bmj* 2010;340:c924. doi: 10.1136/bmj.c924 [published Online First: 2010/03/04]
41. Burton MJ, Faal HB, Ramke J, et al. Announcing The Lancet Global Health Commission on Global Eye Health. *Lancet Glob Health* 2019;7(12):e1612-e13. doi: 10.1016/s2214-109x(19)30450-4 [published Online First: 2019/10/14]

Authors' Contributions: APM, JC, JR and MJB conceived the idea for the review. APM, JR and TB drafted and revised the protocol with suggestions from MJB, JC, JHZ, HF, HT, IJ, NC, AB, TB, MJ, SR, RB, AN, PK and KF. IG constructed the search.

Competing interests: None declared.

Funding Statement: MJB is supported by the Wellcome Trust (207472/Z/17/Z). JR is a Commonwealth Rutherford Fellow, funded by the UK government through the Commonwealth Scholarship Commission in the UK. The Lancet Global Health Commission on Global Eye Health is supported by The Queen Elizabeth Diamond Jubilee Trust, The Wellcome Trust, Sightsavers International, The Fred Hollows Foundation, The SEVA Foundation, The British Council for the Prevention of Blindness and Christian Blind Mission.

Data sharing statement: Data generated from this review will be available upon reasonable request from Ana Patricia Marques (Patricia.Marques@lshtm.ac.uk).

For peer review only

ANNEX 1 - PRISMA-P 2015 CHECKLIST

This checklist has been adapted for use with protocol submissions to *Systematic Reviews* from Moher D et al: Preferred reporting items for systematic review and meta-analysis protocols (PRISMA-P) 2015 statement. *Systematic Reviews* **2015** 4:1

Section/topic	#	Checklist item	Information reported Yes	Information reported No	Line number(s)
**ADMINISTRATIVE INFORMATION**					
**Title**					
Identification	1a	Identify the report as a protocol of a systematic review	☒	☐	3 to 4 (page 1)
Update	1b	If the protocol is for an update of a previous systematic review, identify as such	☐	☒	
**Registration**	2	If registered, provide the name of the registry (e.g., PROSPERO) and registration number in the Abstract	☒	☐	54 (page 3)
**Authors**					
Contact	3a	Provide name, institutional affiliation, and e-mail address of all protocol authors; provide physical mailing address of corresponding author	☒	☐	10 to 59 (page 1)<br>3 to 13 (page 2)
Contributions	3b	Describe contributions of protocol authors and identify the guarantor of the review	☒	☐	33 to 37 (page 15)
**Amendments**	4	If the protocol represents an amendment of a previously completed or published protocol, identify as such and list changes; otherwise, state plan for documenting important protocol amendments	☐	☒	
**Support**					
Sources	5a	Indicate sources of financial or other support for the review	☒	☐	40 to 49 (page 15)
Sponsor	5b	Provide name for the review funder and/or sponsor	☐	☒	
Role of sponsor/funder	5c	Describe roles of funder(s), sponsor(s), and/or institution(s), if any, in developing the protocol	☐	☒	
**INTRODUCTION**					
**Rationale**	6	Describe the rationale for the review in the context of what is already known	☒	☐	6 to 57 (page 5)<br>3 to 40 (page 6)
**Objectives**	7	Provide an explicit statement of the question(s) the review will address with reference to participants, interventions, comparators, and outcomes (PICO)	☒	☐	44 to 49 (page 6)<br>24 to 57 (page 8)

For peer review only

Section/topic	#	Checklist item	Information reported: Yes	Information reported: No	Line number(s)
**METHODS**					
**Eligibility criteria**	8	Specify the study characteristics (e.g., PICO, study design, setting, time frame) and report characteristics (e.g., years considered, language, publication status) to be used as criteria for eligibility for the review	☒	☐	28 to 60 (page 7)<br>3 to 57 (page 8)
**Information sources**	9	Describe all intended information sources (e.g., electronic databases, contact with study authors, trial registers, or other grey literature sources) with planned dates of coverage	☒	☐	3 to 26 (page 7)
**Search strategy**	10	Present draft of search strategy to be used for at least one electronic database, including planned limits, such that it could be repeated	☒	☐	3 to 49<br>Annex 2
STUDY RECORDS					
Data management	11a	Describe the mechanism(s) that will be used to manage records and data throughout the review	☒	☐	56 to 59 (page 9)
Selection process	11b	State the process that will be used for selecting studies (e.g., two independent reviewers) through each phase of the review (i.e., screening, eligibility, and inclusion in meta-analysis)	☒	☐	54 to 60 (page 9)<br>2 (page 10)
Data collection process	11c	Describe planned method of extracting data from reports (e.g., piloting forms, done independently, in duplicate), any processes for obtaining and confirming data from investigators	☒	☐	2 to 19 (page 10)
**Data items**	12	List and define all variables for which data will be sought (e.g., PICO items, funding sources), any pre-planned data assumptions and simplifications	☒	☐	24 to 55 (page 10)
**Outcomes and prioritization**	13	List and define all outcomes for which data will be sought, including prioritization of main and additional outcomes, with rationale	☒	☐	7 to 46 (page 9)
**Risk of bias in individual studies**	14	Describe anticipated methods for assessing risk of bias of individual studies, including whether this will be done at the outcome or study level, or both; state how this information will be used in data synthesis	☒	☐	42 to 60 (page 11)
DATA					
**Synthesis**	15a	Describe criteria under which study data will be quantitatively synthesized	☐	☒	

For peer review only

Section/topic	#	Checklist item	Information reported Yes	No	Line number(s)
	15b	If data are appropriate for quantitative synthesis, describe planned summary measures, methods of handling data, and methods of combining data from studies, including any planned exploration of consistency (e.g., I^2, Kendall's tau)	☐	☒	
	15c	Describe any proposed additional analyses (e.g., sensitivity or subgroup analyses, meta-regression)	☒	☐	51 to 60 (page 11)
	15d	If quantitative synthesis is not appropriate, describe the type of summary planned	☒	☐	14 to 16 (page 12)
**Meta-bias(es)**	16	Specify any planned assessment of meta-bias(es) (e.g., publication bias across studies, selective reporting within studies)	☒	☐	47 to 49 (page 11)
**Confidence in cumulative evidence**	17	Describe how the strength of the body of evidence will be assessed (e.g., GRADE)	☒	☐	3 to 15 (page 10)

For peer review only

ANNEX 2 - SEARCH STRATEGIES

MEDLINE (Ovid)

1. "Global Burden of Disease"/
2. "costs and cost analysis"/
3. cost-benefit analysis/
4. "cost of illness"/
5. health care costs/
6. "Health Services Needs and Demand"/ec, sn [Economics, Statistics & Numerical Data]
7. Health Care Surveys/ec, sn [Economics, Statistics & Numerical Data]
8. Health Expenditures/ec, sn [Economics, Statistics & Numerical Data]
9. Health Resources/ec, sn [Economics, Statistics & Numerical Data]
10. Global Health/ec, sn [Economics, Statistics & Numerical Data]
11. ((global or economic) adj2 burden).tw.
12. ((cost or costs) adj2 (benefit or analysis or illness or direct or indirect or Intangible)).tw.
13. Efficiency/
14. Absenteeism/
15. Presenteeism/
16. productivity.tw.
17. "Severity of Illness Index"/ec [Economics]
18. Employment/ec [Economics]
19. Sick Leave/ec, sn [Economics, Statistics & Numerical Data]
20. (absenteeism or presenteeism or productivity).tw.
21. Caregivers/ec, sn [Economics, Statistics & Numerical Data]
22. or/1-21
23. exp eye diseases/
24. exp vision disorders/
25. ((vision or visual$) adj2 (impair$ or loss or disorder)).tw.
26. (cataract$ or glaucoma or macula$ degeneration).tw.
27. (diabetic retinopathy or refractive error$ or trachoma or corneal opacity).tw.
28. or/23-27
29. 22 and 28
30. limit 29 to yr="2000 -Current"

CRD database

The CRD database will be searched using the following MeSH terms:

MeSH DESCRIPTOR Eye Diseases EXPLODE ALL TREES

MeSH DESCRIPTOR Vision Disorders EXPLODE ALL TREES

For peer review only
